# Advances in Dental Implants, Tissue Engineering and Prosthetic Materials

**DOI:** 10.3390/ma16175871

**Published:** 2023-08-28

**Authors:** Marco Tatullo, Giuseppina Ambrogio, Gilberto Sammartino

**Affiliations:** 1 Department of Translational Biomedicine and Neuroscience, University of Bari “Aldo Moro”, 70124 Bari, Italy; 2 Department of Mechanical, Energy and Management Engineering, University of Calabria, 87036 Arcavacata di Rende, Italy; giuseppina.ambrogio@unical.it; 3 Department of Neurosciences, Reproductive and Odontostomatological Sciences, Postgraduate School of Oral Surgery, University “Federico II” of Naples, Via S. Pansini 5, 80131 Naples, Italy; gilberto.sammartino@unina.it

Scientific research has achieved numerous milestones in the field of materials applied to medicine for biomedical prosthetics. In the early 1980s, the primary goal in the field of materials was to achieve biointegration, and only in the 2000s did we begin to understand the role of biomaterials in tissue regeneration. Over the past 10 years, techniques in tissue engineering, surface functionalization, and biomimicry have been developed to stimulate self-regeneration processes, rendering biomaterials and bioprosthetics capable of enhancing biological and clinical conditions at the implantation site, thereby improving the quality and speed of healing processes. The concept of biomaterials has often been linked to the function of scaffolds because of their ability to interact with their local environment; the multipotency of a scaffold [1] is a natural declination of the bioactivity of a biomaterial as an interface among different tissues, safely interacting with each type of tissue and promoting repair/regeneration in physiological way. In this Special Issue, there is a great interest in so-called smart-materials which are able to plastically customize their support to tissue regeneration based on local clinical/biological conditions. Nonetheless, novel biomaterials and comparative studies with well-established protocols [2] are also interesting because of their contributions to the advancement of knowledge that may be useful in facing several yet-unresolved clinical challenges. Other factors to be considered are the biological effectors in prosthetic rehabilitation: dental-derived mesenchymal stem cells, the extracellular matrix, and exosomes can interact with the environment, ameliorating inflammatory conditions and improving the regenerative processes [3]; there are also other osteogenic factors, such as platelets concentrates [4,5].

Manufacturing processes are also deeply conditioned by the behavior of the materials; stimuli-responsive materials, such as metamaterials, may undergo geometric transformations, showing exceptional dynamic properties and functionalities [6]. In bone tissue engineering, the skills of metamaterials can provide more osseointegration-friendly environments in defects with non-linear geometry [7] or may significantly improve the adaptability of an implant to the primary bone tissue [8].

In conclusion, advances in dental implants, tissue engineering and prosthetic materials are highly needed by clinicians as patients’ expectations have been increasingly raised and achieving clinical performance and aesthetic results is an urgent requirement. Novel materials must merge biological requirements and a friendly manufacturing process that must be increasingly customizable to meet clinical/aesthetical criteria.

The challenge is to completely renovate implant surgery so as to create new opportunities in the dental implantology of the next generation, making the process even more biomimetic and “smart”.
